# Amiodarone-induced granulomatous lung injury mimicking organizing pneumonia: a case report

**DOI:** 10.3389/fphar.2026.1848041

**Published:** 2026-07-15

**Authors:** Alifeila Aili, Hui Deng, Haopeng Zhang, Weiwei Wang, Lei Pan

**Affiliations:** Department of Respiratory and Critical Care Medicine, Emergency and Critical Care Medical Center, Beijing Shijitan Hospital, Capital Medical University, Beijing, China

**Keywords:** amiodarone pulmonary toxicity, case report, drug-induced lung injury, granulomatous lung injury, organizing pneumonia, reversed halo sign

## Abstract

**Background:**

Amiodarone-induced pulmonary toxicity (APT) has a broad clinical spectrum, and its radiologic and histopathologic appearances vary considerably. Granulomatous lung injury, however, is rarely described and may be confused with organizing pneumonia (OP) or infection.

**Case presentation:**

An elderly man who had been receiving 5-month amiodarone therapy developed a 3-week history of pleuritic chest pain and progressive dyspnea. Chest computed tomography (CT) demonstrated bilateral ground-glass opacities and subpleural-predominant consolidations, with scattered reversed halo signs raising the possibility of an OP-like pattern. A positive serum Cryptococcal antigen (CrAg) result obtained at an outside hospital led to empiric antifungal therapy, but the patient did not improve. After admission, bronchoalveolar lavage (BAL) revealed lymphocytosis, and metagenomic testing did not detect Cryptococcus or other pathogens; fungal stains on biopsy specimens were also negative. Percutaneous lung biopsy showed focal non-necrotizing granulomas with prominent eosinophilic inflammation. After discontinuation of amiodarone and initiation of systemic corticosteroid therapy, his symptoms improved rapidly and follow-up imaging demonstrated interval regression.

**Conclusion:**

This case illustrates that an OP-like CT pattern may mask an uncommon granulomatous phenotype of amiodarone-related lung injury. A positive fungal biomarker should therefore be weighed against the microbiological work-up, tissue findings, medication history, and treatment response before infection is accepted as the final diagnosis.

## 1 Introduction

Amiodarone is a class III antiarrhythmic agent that remains widely used for atrial fibrillation and ventricular arrhythmias because of its broad efficacy (Mason, 1987; Vassallo and Trohman, 2007). However, amiodarone is also associated with extra-cardiac adverse effects, and pulmonary toxicity is a well-recognized complication of long-term therapy. Amiodarone-induced pulmonary toxicity (APT) encompasses heterogeneous clinical courses and radiologic patterns, including nonspecific interstitial pneumonia, eosinophilic pneumonia, organizing pneumonia, acute respiratory distress syndrome, and lipoid pneumonia. The reported incidence of pulmonary toxicity among long-term users is approximately 1%–5% (Papiris et al., 2010; Feduska et al., 2021; Kwok et al., 2022; Al-Shammari et al., 2016; Jackevicius et al., 2011). Reports of biopsy-proven amiodarone-related granulomatous lung injury are rare, and there are no specific biomarkers. In clinical practice, the diagnosis of amiodarone-induced granulomatous lung injury often depends on reconstructing a detailed history of drug exposure alongside careful interpretation of radiologic findings, bronchoalveolar lavage profiles, and tissue pathology, while alternative causes such as infection or other interstitial lung diseases must be systematically excluded (Papiris et al., 2010; Rosen, 2022; Roden and Camus, 2018). Here, we report a patient with an OP-like CT pattern and biopsy-proven non-necrotizing granulomas with eosinophil-rich inflammation after 5 months of oral amiodarone therapy, illustrating a key diagnostic pitfall and important differential considerations.

## 2 Case presentation

A 70-year-old male was admitted with a 3-week history of pleuritic chest pain and progressive dyspnea. The pain was sharp, localized to the left lower chest, and worsened with inspiration. Exertional dyspnea occurred during routine activities. He had been evaluated at an outside institution, where bronchoscopy was performed. The bronchoalveolar lavage (BAL) report described a macrophage- and lymphocyte-predominant profile, with a CD4/CD8 ratio of 1.8. A serum Cryptococcal antigen (CrAg) test was reported as positive; however, the specimen type, assay method, and titer were not available in the provided records. Brain magnetic resonance imaging showed no intracranial focus of infection. He received empiric antibacterial therapy and a short course of oral fluconazole, without meaningful improvement.

Medical history and exposures: The patient had a 20-year history of hypertension treated with perindopril. He had coronary artery disease with prior percutaneous coronary intervention and had been taking atorvastatin 20 mg daily. He also had an 8-year history of paroxysmal atrial fibrillation and had received amiodarone (200 mg/day) and rivaroxaban continuously for the past 5 months. He denied any drug or food allergies, smoking history, bird exposure, pet ownership, or relevant occupational exposures. No recent high-humidity environmental exposure was reported.

Physical Examination on Admission: On initial assessment, the patient was afebrile (temperature 36.5 °C), with an irregular rhythm at 70 bpm with frequent supraventricular premature beats. Blood pressure was 132/75 mmHg, respiratory rate 16 breaths per minute, and oxygen saturation 94% on room air. Lung auscultation revealed bilateral coarse breath sounds with Velcro-like crackles over the left lung fields. No peripheral edema was present. Abdominal, neurologic, and skin examinations were unremarkable.

Diagnostic Investigations: Arterial blood gas analysis on room air showed PaO2 of 69 mmHg, with a PaO2/FiO2 ratio of 329 mmHg and an elevated alveolar-arterial oxygen gradient (38 mmHg). Serum C-reactive protein (CRP) was mildly elevated (23.22 mg/L; reference range <8 mg/L); procalcitonin was normal (0.034 ng/mL; reference range <0.05 ng/mL). Taken together, the normal procalcitonin level and the lack of clinical response to empiric antibiotics made typical bacterial pneumonia less likely. The serum Cryptococcal capsular antigen test was positive whereas repeat fungal cultures (sputum and blood) were negative. The total level of serum IgE was reportedly normal (34.10 IU/mL); complement (total complement >60 U/mL, C3 1.43 g/L) and cytokines levels (IL-6 17.01 pg/mL, IL-8 34.11 pg/mL) were elevated—indicating a proinflammatory state. Complete blood count showed a normal white blood cell count (8.26 × 10^9^/L) but mild lymphopenia (1.06 × 10^9^/L). Lymphocyte subset analysis revealed a T-helper cell (CD4^+^ T-cell) count of 368/µL (35.97%), a T-suppressor cell (CD8^+^T-cell) count of 164/µL (16.06%), resulting in a T-helper/T-suppressor cell (CD4^+^/CD8^+^) ratio of 2.2. Antinuclear antibody (ANA), ANCA-associated antibodies, and tumor markers (carcinoembryonic antigen [CEA], cytokeratin-19 fragment [CYFRA21-1], and neuron-specific enolase [NSE]) were negative, ruling out autoimmune disease and malignancy-related lung lesions. Echocardiography demonstrated left atrial enlargement and impaired left ventricular diastolic function. Pulmonary function tests detected a restrictive ventilatory defect: vital capacity (VC) 2.44 L (59.7% of predicted), forced expiratory volume in 1 s (FEV1) 1.96 L (64.3% of predicted), forced expiratory volume in 1 s/forced vital capacity (FEV1/FVC) ratio 80.27%. The diffusing capacity for carbon monoxide (DLCO) was significantly reduced to 3.80 mmol/min/kPa (42.8% of predicted). Fractional exhaled nitric oxide (FeNO) was elevated (53 ppb), suggesting airway inflammation.

Pre-admission high-resolution computed tomography (HRCT) of the chest, acquired with 1.25-mm thin-section reconstructions using a bone/high-spatial-frequency algorithm, showed mild thickening of the bronchovascular bundles and bronchial walls, with mild multifocal distal bronchiolectasis. Multifocal patchy and linear ground-glass opacities and mildly consolidative opacities were present in both lungs, predominantly in the subpleural regions, and some lesions were accompanied by interlobular septal thickening. Mild bilateral pleural thickening was also noted (Figure 1). This subpleural-predominant distribution of ground-glass and consolidative abnormalities was considered compatible with an organizing pneumonia-like pattern. However, because the serum cryptococcal antigen test was positive, a concurrent infection was initially suspected, and fluconazole was continued for a short period during further diagnostic work-up (400 mg once daily for 6 days). No obvious hyperattenuation was identified within the pulmonary consolidative lesions on the available non-contrast chest HRCT images. Because abdominal CT was not performed, hepatic or splenic attenuation could not be assessed; however, abdominal ultrasonography showed no hepatosplenomegaly.

**FIGURE 1:**
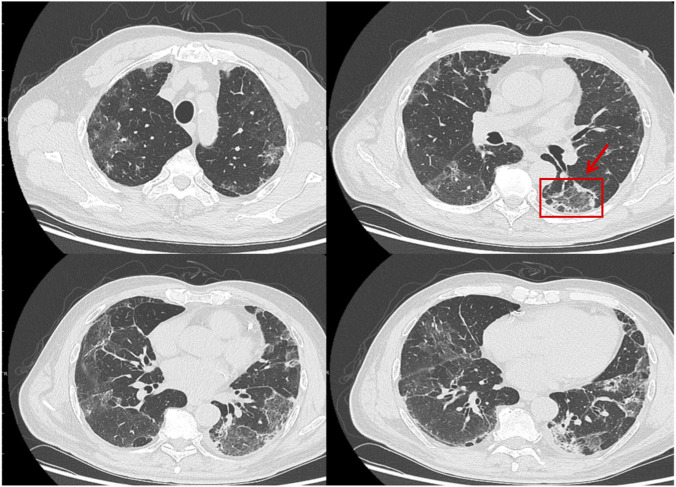
Admission HRCT findings. 1.25-mm thin-section lung-window images reconstructed using a bone algorithm show multifocal patchy and linear ground-glass opacities and mildly consolidative opacities in both lungs, predominantly in the subpleural regions. A representative reversed halo sign is highlighted by the red arrow and box, supporting an organizing pneumonia-like pattern.

To clarify the etiology, bronchoscopy with bronchoalveolar lavage (BAL) was performed. BAL fluid analysis revealed a lymphocytic predominance (42%), with 45% macrophages, 13% neutrophils, and no eosinophils. Metagenomic sequencing of BAL fluid was negative for any other pathogenic bacteria, fungi, viruses, or mycobacteria. Moreover, ultrasound-guided percutaneous lung biopsy revealed widened alveolar septa, focal interstitial fibrous proliferation, and well-formed non-necrotizing granulomas composed of epithelioid histiocytes, mainly located within the pulmonary interstitium. The granulomatous lesions were accompanied by eosinophil-rich inflammatory infiltration (Figure 2). Special stains (Gomori methenamine silver [GMS] and periodic acid-Schiff [PAS]) showed no morphological evidence of fungi or Cryptococcus. The aforementioned pathological morphology does not fully align with the typical manifestations of organizing pneumonia characterized by Masson bodies.

**FIGURE 2:**
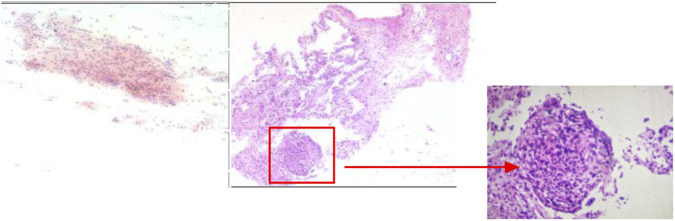
Histopathological examination of lung biopsy (H&E staining). Percutaneous biopsy shows well-formed non-necrotizing granulomas composed of epithelioid histiocytes, mainly located within the pulmonary interstitium, with eosinophil-rich inflammatory infiltration and focal interstitial fibrous proliferation. The granulomatous focus is highlighted by the red box and enlarged in the right panel. Special stains (GMS and PAS) showed no morphologic evidence of fungi, including Cryptococcus.

Infection did not explain the course. The patient failed to improve with empirical antibacterial therapy or a short trial of fluconazole, and bronchoalveolar lavage testing—including metagenomic next-generation sequencing, cultures, and tissue stains—did not identify a pathogen. Other interstitial lung diseases were considered as well. Autoimmune screening was negative, and the history did not suggest an exposure pattern typical of hypersensitivity pneumonitis. Sarcoidosis was also considered because of the non-necrotizing granulomatous inflammation and BAL lymphocytosis. However, chest HRCT showed no obvious bilateral hilar or mediastinal lymphadenopathy. In addition, superficial lymph node ultrasonography and abdominal ultrasonography did not reveal abnormal lymphadenopathy, and no hepatosplenomegaly was observed. Together with the absence of extrapulmonary manifestations on physical examination, these findings made systemic sarcoidosis less likely. Several findings instead pointed toward drug-related lung injury, including 5-month amiodarone exposure, markedly reduced diffusing capacity for carbon monoxide, an organizing pneumonia–like computed tomography pattern with reversed halo signs, bronchoalveolar lavage lymphocytosis, and biopsy-proven non-necrotizing granulomatous inflammation with eosinophil-rich infiltration. On balance, amiodarone-associated granulomatous lung injury was favored after exclusion of other likely causes.

Treatment and Outcome: Amiodarone was immediately discontinued and intravenous methylprednisolone therapy was initiated (initial dose 60 mg/day, reduced to 40 mg/day after 3 days), supplemented with acid suppression and calcium supplementation as supportive measures. Symptoms improved within 48 h of corticosteroid initiation, and chest HRCT obtained before discharge showed marked interval improvement (Figure 3). Upon discharge, oral prednisone (40 mg/day) was prescribed, with a weekly dose reduction of 4 mg and scheduled outpatient follow-up.

**FIGURE 3:**
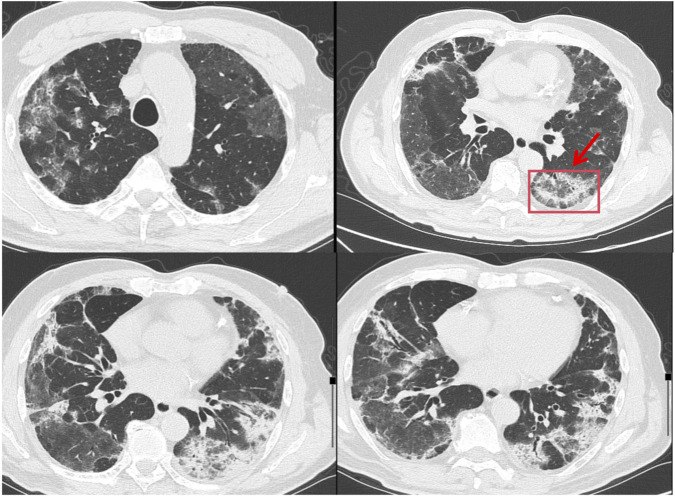
Pre-discharge HRCT findings. 1.25-mm thin-section lung-window images reconstructed using a bone algorithm show marked regression of the previously noted subpleural ground-glass and consolidative opacities. The red arrow and box indicate the corresponding region where the reversed halo-like lesion shown in Figure 1 had substantially regressed.

After discharge, the patient continued oral glucocorticoid therapy and was transitioned to prednisone 32 mg/day. At the 1-month follow-up visit, symptoms remained improved and repeat imaging demonstrated further radiologic resolution (Figure 4), indicating a favorable therapeutic response. Key clinical events are summarized below (Table 1).

**FIGURE 4:**
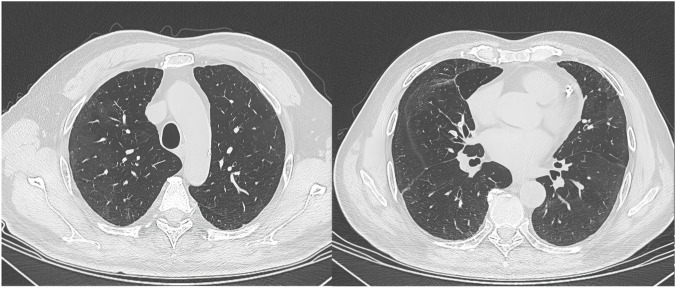
Follow-up HRCT at 1 month post-discharge. 1.25-mm thin-section lung-window images reconstructed using a bone algorithm show further radiologic resolution of the prior infiltrates during glucocorticoid tapering.

**TABLE 1:** Key clinical events.

Time point	Event
8 years before admission	Diagnosed with paroxysmal atrial fibrillation
5 months before admission	Started amiodarone therapy at 200 mg/day and rivaroxaban for paroxysmal atrial fibrillation
3 weeks before admission	Onset of pleuritic chest pain and progressive dyspnea
Outside hospital	Bronchoscopy performed; serum CrAg reported positive; empiric antibiotics and short course of fluconazole with poor response
Hospital day 0–1	Admission evaluation and CT suggestive of organizing pneumonia-like pattern; BAL obtained; BAL metagenomics negative; percutaneous lung biopsy performed
Hospital day 1–3	Amiodarone discontinued; intravenous methylprednisolone initiated with rapid symptomatic improvement
Before discharge	Symptoms improved; chest CT demonstrated marked interval regression; discharged on oral prednisone with taper plan
1 month after discharge	Further radiologic resolution on follow-up CT with continued glucocorticoid tapering

## 3 Discussion

Amiodarone-induced pulmonary toxicity (APT) is a clinically important adverse drug reaction that can progress to respiratory failure and, in severe cases, death (Roden and Camus, 2018; Mason, 1987; Vassallo and Trohman, 2007; Papiris et al., 2010; Feduska et al., 2021). Its clinical manifestations are often nonspecific and may follow acute, subacute, or chronic courses with heterogeneous presentations. Diagnosis is largely one of exclusion and requires careful integration of medication exposure, imaging findings, BAL profiles, and histopathology, while alternative causes such as infection, cardiogenic pulmonary edema, and other interstitial lung diseases are systematically ruled out (Papiris et al., 2010; Rosen, 2022; Roden and Camus, 2018). In this case, the patient presented with diffuse pulmonary infiltrates accompanied by reversed halo signs, and biopsy demonstrated focal non-necrotizing granulomas with eosinophil-rich inflammation, representing a rare and easily overlooked manifestation of APT.

Pulmonary granulomas are organized inflammatory structures composed mainly of epithelioid cells/macrophages, which may or may not be accompanied by necrosis, multinucleated giant cells, and surrounding inflammatory infiltrates (Rosen, 2022). The etiologic spectrum of pulmonary granulomatous lesions is broad; initial evaluation should distinguish infectious causes (e.g., mycobacteria and fungi) from noninfectious entities (e.g., sarcoidosis, exposure-related disorders, immune-mediated diseases, and drug reactions) (Rosen, 2022; Franquet et al., 2021). In drug-induced lung injury, granulomas are often poorly formed to moderately developed and may coexist with bronchiolitis and interstitial inflammation, producing morphological overlap with hypersensitivity pneumonitis (HP) (Roden and Camus, 2018; Rambhia et al., 2019).

Granulomatous inflammation is not a common dominant pathologic pattern in amiodarone-related pulmonary toxicity, which more often manifests as interstitial pneumonia, OP, eosinophilic pneumonia, acute lung injury, or lipoid pneumonia. Granulomatous lung reactions are more commonly described with agents such as methotrexate, tumor necrosis factor-alpha (TNF-alpha) antagonists, interferons, and immune checkpoint inhibitors (Roden and Camus, 2018; Rambhia et al., 2019; Imokawa et al., 2000). Nevertheless, amiodarone pulmonary toxicity is thought to involve both direct cytotoxicity of the drug and immune-mediated inflammatory responses. Previous reports have also documented amiodarone-induced hypersensitivity pneumonitis, suggesting its potential to trigger cell-mediated immune mechanisms (Papiris et al., 2010; Akoun et al., 1984). Furthermore, case reports of amiodarone-induced granulomatous reactions in extrapulmonary organs (such as the liver, bone marrow, and lymph nodes) also suggest its potential immunorelevance (Harrison and Elias, 1993; Erie et al., 2010; Moran and Manoharan, 2002; Boutros et al., 2000). In our patient, the temporal relationship with amiodarone exposure, exclusion of alternative causes, and rapid clinical and radiologic improvement after amiodarone withdrawal and corticosteroid therapy supported the causal inference of drug-induced lung injury. Although foamy macrophages and vacuolated pneumocytes were not identified in the biopsy specimen, this finding alone was not sufficient to rule out APT. The tissue was obtained by ultrasound-guided percutaneous needle biopsy, and the limited sampling area may not have captured the full spectrum of amiodarone-related histologic changes. The well-formed interstitial non-necrotizing granulomas and eosinophil-rich inflammation therefore needed to be interpreted in this broader clinical context, rather than as isolated histological findings.

A major diagnostic pitfall in this case was the positive serum cryptococcal antigen result obtained at the outside hospital. The exact reason for this positive result remains uncertain because the specimen type, assay method, and antigen titer were not available. Possible explanations include a low-titer false-positive reaction, nonspecific assay interference, problems related to sample handling or testing procedures, contamination, or cross-reactivity with non-Cryptococcus organisms. Cryptococcosis was considered unlikely in our patient because empirical antifungal therapy produced no meaningful improvement, BAL metagenomic sequencing did not detect Cryptococcus or other fungal pathogens, fungal cultures were negative, and GMS and PAS staining of the biopsy specimen showed no morphologic evidence of fungi. The rapid clinical and radiologic response after amiodarone withdrawal and corticosteroid therapy further supported a noninfectious drug-induced inflammatory process. This case highlights the need to interpret fungal biomarkers in the context of microbiological, histopathological, and clinical evidence.

Radiologically, the bilateral subpleural-predominant ground-glass opacities, mildly consolidative opacities, and reversed halo-like lesion were most compatible with an OP-like pattern. NSIP-like involvement was also considered because amiodarone pulmonary toxicity can present with an NSIP pattern, and this patient had bilateral ground-glass opacities together with BAL lymphocytosis. However, the HRCT pattern was not typical of NSIP. The abnormalities were multifocal and patchy, with subpleural and peribronchovascular predominance, mildly consolidative opacities, and a reversed halo-like lesion, rather than a relatively symmetric and homogeneous ground-glass or fine reticular pattern. In addition, the biopsy demonstrated non-necrotizing granulomatous inflammation with eosinophil-rich infiltration, which is not a typical histopathological feature of NSIP.

The histopathologic findings also did not fully align with classic OP, which is usually characterized by intra-alveolar fibroblastic plugs, or Masson bodies. Instead, the percutaneous biopsy showed non-necrotizing granulomatous inflammation with eosinophilic infiltration. The increased lymphocyte proportion in BAL fluid suggested an immune-mediated inflammatory response, which may be seen in HP or drug-induced hypersensitivity pneumonitis. However, the CD4/CD8 ratio alone cannot exclude HP and should be interpreted together with exposure history, HRCT features, and the overall histological pattern (Watts and Grammer, 2019; Rea et al., 2024). In this patient, the absence of a relevant environmental or occupational exposure history and the lack of typical HRCT features such as mosaic attenuation or air trapping made HP less likely.

In differential diagnosis, in addition to infection, conditions such as sarcoidosis-like reactions, drug-induced granulomatous lung diseases, and immune-related disorders should also be considered (Rosen, 2022; Roden and Camus, 2018; Franquet et al., 2021). The combination of non-necrotizing granulomas, BAL lymphocytosis and subpleural abnormalities also raised the differential diagnosis of sarcoidosis or a drug-induced sarcoid-like reaction. However, the overall clinicopathological picture was not typical of sarcoidosis. Chest HRCT showed no obvious bilateral hilar or mediastinal lymphadenopathy, and ultrasound evaluation showed no abnormal superficial or abdominal lymphadenopathy or hepatosplenomegaly. No extrapulmonary features suggestive of systemic sarcoidosis were identified on physical examination. Moreover, the eosinophil-rich inflammatory background, temporal association with amiodarone exposure, negative microbiological work-up, and rapid clinical and radiologic response after amiodarone withdrawal and corticosteroid treatment favored amiodarone-associated granulomatous lung injury. Although a drug-induced sarcoid-like reaction could not be completely excluded on histology alone, the overall clinical course supported amiodarone-related lung injury as the most likely diagnosis.

This case still has some limitations. Surgical lung biopsy was not performed, and the limited size of the percutaneous biopsy specimen may not have captured the full histological spectrum of the disease. In addition, the positive cryptococcal antigen result from the outside hospital could not be fully characterized because the assay type, specimen source, and titer were unavailable. Despite these limitations, the integrated clinical, radiologic, microbiological, and pathological findings, together with the favorable response to amiodarone discontinuation and corticosteroid therapy, strongly supported the final diagnosis.

The cornerstone of APT management is prompt discontinuation of the causative drug. Corticosteroids are commonly used when clinically significant inflammatory lung injury is present and active infection has been reasonably excluded. In this case, intravenous methylprednisolone followed by oral prednisone tapering was associated with rapid symptomatic improvement and sustained radiologic regression, consistent with previous reports of favorable responses to corticosteroid therapy in APT (Terzo et al., 2020). For patients on long-term amiodarone therapy, it is recommended to establish baseline chest imaging and pulmonary function (particularly diffusion capacity) assessments at the initiation of treatment, with regular follow-up evaluations to facilitate early identification of subclinical pulmonary injury (Papiris et al., 2010).

## 4 Conclusion

This case describes a rare granulomatous lung injury associated with 5 months of oral amiodarone therapy. The main diagnostic challenge arose from the coexistence of an organizing pneumonia-like radiologic pattern, non-necrotizing granulomatous inflammation on lung biopsy, and a misleading positive serum cryptococcal antigen result. The diagnosis of amiodarone-associated granulomatous lung injury was supported by the medication history, exclusion of infectious and autoimmune causes, BAL cytology and microbiological testing, histopathology, and the rapid response to amiodarone withdrawal and corticosteroid therapy. In patients with diffuse lung disease and a poor response to anti-infective therapy, clinicians should carefully review medication exposure and consider rare granulomatous phenotypes of amiodarone-induced pulmonary toxicity in the differential diagnosis.

## Data Availability

The original contributions presented in the study are included in the article/supplementary material, further inquiries can be directed to the corresponding authors.
